# Do ethnic differences in cord blood leptin levels differ by birthweight category? Findings from the Born in Bradford cohort study

**DOI:** 10.1093/ije/dyt225

**Published:** 2013-11-29

**Authors:** Jane West, John Wright, Lesley Fairley, Naveed Sattar, Peter Whincup, Debbie A Lawlor

**Affiliations:** ^1^Bradford Institute for Health Research, Bradford Teaching Hospitals NHS Foundation Trust, Bradford, UK, ^2^Institute of Cardiovascular and Medical Sciences, BHF Glasgow Cardiovascular Research Centre, University of Glasgow, Glasgow, UK, ^3^Division of Population Health Sciences and Education, University of London, London, UK and ^4^MRC Centre for Causal Analyses in Translational Epidemiology, School of Social and Community Medicine, University of Bristol, Bristol, UK

**Keywords:** Birthweight, birth size, cord blood leptin, South Asian

## Abstract

**Background** There is evidence that South Asian individuals have higher fat mass for a given weight than Europeans. One study reported that the greater fatness for a given birthweight may increase with increasing birthweight, suggesting that any attempt to increase mean birthweight in South Asians would markedly increase their fatness.

**Objective** Our objective was to examine whether differences in cord leptin values between White British and Pakistani infants vary by birthweight category.

**Method** We examined the difference in cord leptin levels between 659 White British and 823 Pakistani infants recruited to the Born in Bradford cohort study, by clinical categories and thirds of the birthweight distribution.

**Results** Pakistani infants had a lower mean birthweight but higher cord leptin levels than White British infants [ratio of geometric mean (RGM) of cord leptin adjusted for birthweight = 1.36 (95% CI 1.26, 1.46)]. Birthweight was positively associated with cord leptin levels in both groups, with no evidence that the regression lines in the two groups diverged from each other with increasing birthweight. The relative ethnic difference in cord leptin was similar in low (<2500 g), normal and high (≥4000 g) birthweight infants (*P*-value for interaction = 0.91). It was also similar across thirds of the birthweight distribution [RGM (95% CI) in lowest, mid and highest thirds were 1.37 (1.20, 1.57), 1.36 (1.20, 1.54) and 1.31 (1.16, 1.52), respectively, *P*-interaction = 0.51].

**Conclusions** We found marked differences in cord leptin levels between Pakistani and White British infants but no evidence that this difference increases with increasing birthweight.

## Introduction

Infants of South Asian origin have been found to have consistently lower birthweights than those of European origin.^1–3^ These differences are present even among infants born in Western populations.^4–6^ Using data from the Born in Bradford (BiB) cohort, we have recently shown that the magnitude of the birthweight difference between UK-born Pakistani origin and White British infants is the same whether both, one or neither parent were born in the UK.^7^ However, we have also found that, for a given birthweight, Pakistani origin infants had higher cord leptin levels, suggesting that they were more adipose. This finding suggests that the thin-fat insulin resistant phenotype previously described in adults,^8^ children,^9^^,^^10^ and infants^11^ might also be present at birth. It also raises the possibility that any attempts to increase birthweight in South Asian infants might inadvertently worsen health by increasing adiposity. If the ethnic difference in adiposity for a given birthweight increases as birthweight increases, then this potential adverse effect of attempts to increase South Asian birthweight could be particularly detrimental. To our knowledge, only one study has drawn attention to this possibility, and it suggested that the pattern of greater relative adiposity (as assessed by arm fat index in that study) among large for gestational age infants (≥90th percentile) born in India compared with large for gestational age North American infants was greater than in normal for gestational age birthweights^12^ However, in that study there were only 16 infants (6 Indian and 10 North American) in the large for gestational age category and there was no statistical evidence of a difference in the association of ethnicity with arm fat index between the large and normal for gestational age groups.

The aim of this study was to examine whether the difference in cord leptin, a valid marker for percent fat mass, between Pakistani and White British infants increases with increasing birthweight.

## Method

### Participants

The BiB study is a prospective birth cohort study that recruited women during pregnancy and has followed them, their infants and their partners into childhood. To be eligible for the study, women had to attend booking clinic between March 2007 and December 2010 and be booked to give birth in the city of Bradford. Full details of the study methodology have been previously reported.^13^ Women were recruited at their oral glucose tolerance test (OGTT) appointment. All women booked for delivery in Bradford are offered a 75-g OGTT (comprising fasting and 2-h post-load samples) at around 26–28 weeks gestation. Women who attended this appointment and agreed to take part in the study consented to the abstraction and use of data from their obstetric medical records, had their height and weight measured and completed an interviewer administered questionnaire. The questionnaire included questions relating to ethnicity, social and economic circumstances, smoking, alcohol, diet, education and employment. Interviews were conducted in a range of South Asian languages (including Mirpuri, Bengali, Punjabi). Ethics approval for the study was granted by Bradford National Health Service Research Ethics Committee (ref 06/Q1202/48). Cord blood samples for the assessment of leptin levels were collected from infants born between October 2008 and October 2009. The eligible cohort for this study was therefore the 1721 mother-live offspring pairs where the mother delivered at term and belonged to the subgroup with cord leptin measurements. We excluded infants born to parents of ethnic origin other than White British or Pakistani (*N* = 239). Thus 1482 participants were included (659 White British and 823 Pakistani).

### Exposure measurement

Ethnicity was self-reported at the mother’s questionnaire interview and based on UK Office of National Statistics guidance. For all infants in this study, both parents had the same ethnic origin (either both Pakistani origin or both White British origin). Among Pakistani origin infants, 9.7% had parents who were both UK born, 58.8% had one UK-born parent and 22% had parents who were both born in South Asia. Birthweight was abstracted from the medical records and in all participants was recorded immediately following birth by midwives.

### Outcome measures

Cord blood samples were obtained at delivery by the attending midwife. Samples were refrigerated at 4°C in EDTA tubes until collected by laboratory staff within 12 h. Samples were then spun, frozen and stored at −80°C. They were transferred to the Biochemistry Department of Glasgow Royal Infirmary for analysis, where leptin was measured by a highly sensitive in-house ELISA with better sensitivity at lower levels than commercial assays as previously described.^13^

### Statistical analyses

All analyses were performed using STATA (version 11). Characteristics of White British and Pakistani origin infants are presented using numbers (%) for categorical characteristics and mean (SD) or median (IQR) for continuously measured variables. To examine whether the ethnic difference (relative to birthweight) in cord leptin differed by birthweight, we present scatter plots and regression lines from the multivariable regression models of the association of birthweight with cord leptin for each ethnic group. If regression lines diverge with increasing birthweight, this would suggest that the ethnic difference increases with increasing birthweight, whereas the opposite would be true if the lines converge or if they remain parallel. This would suggest no effect of birthweight on the ethnic difference in cord leptin levels. We further explored this by comparing the ethnic differences in cord leptin in strata of birthweights and examined evidence for statistical interaction between these strata and ethnicity in relation to cord leptin. We did this stratified analysis in two ways, first using clinical criteria for low (<2500 g), normal (2500−3999 g) and high (4000 g and over) birthweight. Second, by thirds of the birthweight distribution: the former has clinical meaning but the latter has greater statistical power since the sample size in each group is the same. Cord leptin levels had a positively skewed distribution and were natural log transformed to achieve approximate normality of the residuals in the regression models. The resultant coefficients were back transformed to give ratios of geometric means which can be interpreted as percentage differences and have a null value of 1.

## Results

**Figure 1:**
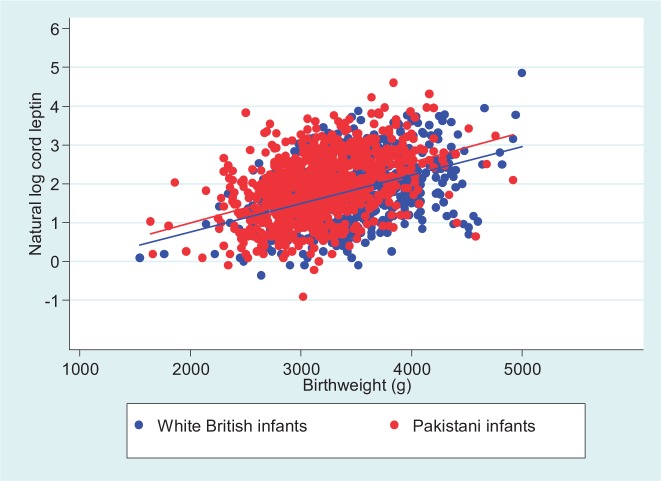
Correlation between birthweight and leptin by ethnic group;r^2^ White British infants = 0.1838, r^2^ Pakistani infants = 0.1806

Table 1 shows the characteristics of the sample by ethnic group. Pakistani infants had a lower mean birthweight [3189 g (SD 450)] compared with White British infants [3458 g (SD 482)] and a higher prevalence of low birthweight (4.9% compared with 1.5% among White British infants) but higher median cord leptin [6.9 (IQR 3.9, 12.2) compared with 6.1 (IQR 3.4, 10.8) among White British infants]. With adjustment for birthweight in the whole cohort cord leptins were on average 36% greater in Pakistani compared with White British origin infants (Table 2). The ethnic difference in cord leptin was similar in low (<2500 g), normal and high (≥4000 g) birthweight infants (*P*-value for interaction = 0.91) (Table 2). It was similar across thirds of the birthweight distribution (*P*-value for interaction = 0.51) (Table 3). The association of birthweight with cord leptin was positive and linear in both ethnic groups, with the regression line for the ethnic groups being parallel across the whole birthweight distribution, further illustrating that the ethnic difference in cord leptin did not vary by birthweight (Figure 1). This pattern was similar for males and females and did not substantively alter when infants of mothers with a diagnosis of gestational diabetes were removed from the analysis (Supplementary Figure 1, available as Supplementary data at *IJE* online).

**Table 1:** Characteristics by ethnic group

Characteristic	White British infants *N* = 659	Pakistani infants *N* = 823	*P*-Value for the ethnic difference
Gender, male *N* (%)	338 (51)	410 (50)	0.573
Birthweight, mean grams (SD)	3458 (482)	3189 (450)	0.000
Gestation at delivery (>37 weeks)^a^	39.5	39.4	0.064
Low birthweight, *N* (%)	10 (1.5)	41 (4.9)	0.000
Cord leptin, median ng/ml (IQR)	6.1 (3.4, 10.8)	6.9 (3.9, 12.2)	0.138

Number (*N*) (%) for categorical variables; mean (SD) or median (IQR) for continuously measured variables. ^a^Completed weeks.

**Table 2:** Differences in cord leptin between Pakistani infants and White British infants by clinical birthweight groups

	Ratio of geometric means comparing Pakistani with White British (95% CI)			
Birthweight	Unadjusted cord leptin	Cord leptin adjusted for gender	Cord leptin adjusted for gender and birthweight	*P*-value for interaction by birthweight^a^
All birthweights	1.10 (1.01 , 1.19)	1.09 (1.00, 1.18)	1.36 (1.26, 1.46)	
Births <2500 g (*N* = 51)	1.90 (1.14, 3.17)	1.88 (1.09, 3.23)	1.62 (0.93, 2.83)	0.90
Births 2500−3999 g (*N* = 1308)	1.17 (1.07, 1.27)	1.18 (1.09, 1.28)	1.36 (1.26, 1.48)	
Births >4000 g (*N* = 123)	1.32 (0.95, 1.84)	1.20 (0.87, 1.66)	1.23 (0.89, 1.69)	

^a^Testing the null hypothesis that the difference in cord leptin between Pakistani and White British infants is the same in each clinical birthweight group in the fully adjusted (gender and birthweight) model.

**Table 3:** Differences in cord leptin between Pakistani and White British infants by thirds of the birthweight distribution

	Ratio of geometric means comparing Pakistani to White British (95% CI)			
Distribution	Unadjusted cord leptin	Cord leptin adjusted for gender	Cord leptin adjusted for gender and birthweight	*P*-value for interaction by birthweight
Lowest third birthweight^a^ (*N* = 509)	1.29 (1.12, 1.49)	1.31 (1.15, 1.51)	1.37 (1.20, 1.57)	0.51
Middle third birthweight^b^ (*N* = 497)	1.30 (1.14, 1.48)	1.34 (1.18, 1.52)	1.36 (1.20, 1.54)	
Highest third birthweight^c^ (*N* = 476)	1.31 (1.13, 1.51)	1.26 (1.10, 1.44)	1.31 (1.16, 1.52)	

^a^Range of birthweights 1540−3100 g.

^b^Range of birthweights 3110−3500 g.

^c^Range of birthweights 3510−5000 g.

## Discussion

There is increasing evidence that South Asians have a characteristic phenotype of proportionately greater adiposity, increased insulin resistance and higher rates of diabetes and cardiovascular disease compared with White European individuals^8^^,^^15^ and recent evidence suggests that this phenotype may be present at birth in UK South Asians.^7^ Thus, efforts to reduce existing ethnic differences in birthweight by increasing South Asian mean birthweight may inadvertently result in greater percent of fat and worsen long-term health outcomes for South Asian infants. Indeed, one previous study suggested that this might be even greater than implied by an independent association of birthweight with fatness and ethnicity with fatness, in South Asian infants compared with White European origin infants, increased with increasing birthweight.^11^ If this is true, then any intervention that effectively increased mean South Asian birthweight could be particularly detrimental to future cardiovascular health. However, that previous study was limited by a relatively small sample size and the comparison of Indian babies born in India (i.e. to women who had lived all their lives in India to the point of their birth of their infants) with American infants born in the USA. It also used arm fat index to compare the difference in adiposity across birthweight categories. Here, we have examined the ethnic difference in cord leptin levels across the entire birthweight distribution and by both clinical categories and thirds of the birthweight distribution, and found no evidence that the ethnic difference in fatness for a given birthweight increases with increasing birthweight. Indeed, when examined by clinical categories, the point estimate suggested that the ethnic difference was, if anything, larger in those in the low birthweight category, but this group included just 51 participants and there was no strong statistical evidence that the ethnic difference in this group differed from that in the normal and large birthweight groups.

To our knowledge, our study is by far the largest to compare fatness between White British and South Asian origin infants resident in the same area and receiving the same antenatal care and using a robust biomarker of body fat at birth (cord leptin). Further, we have examined a possible interaction between ethnicity and birthweight with infant fatness using the whole birthweight distribution and both clinical and statistically more robust categories of birthweight, finding consistent evidence with all of these approaches that the ethnic difference in relative fatness does not increase with increasing birthweight. We have used cord blood leptin as a measure of foetal/birth fat mass. Leptin is found in foetal adipose tissue, and cord blood leptin is strongly related to foetal fat mass, but not to lean mass or other foetal measurements.^16^^,^^17^ Whilst it is known that the placenta secretes leptin, the vast majority of this (at least 95%) is secreted into the maternal circulation, with very little going into the foetal circulation, meaning that cord-blood leptin reflects foetal fat mass, with less than 1% estimated to be from the placenta/maternal circulation.^17^^,^^18^ Furthermore, we found that cord blood leptin explained approximately 18% of the variation in birthweight in our cohort, which is consistent with results of studies that have measured the proportion of neonatal fat composition.^11^ A limitation of this study was the inability to include other South Asian groups (Indian and Bangladeshi) due to relatively small numbers of these groups in the city of Bradford and hence in our cohort. Whereas examining a specific South Asian population by country of birth (i.e. Pakistan) reduces the potential problem of heterogeneity between South Asian groups, it may at the same time limit generalisability to other South Asian populations.

In summary, we have examined the difference in fatness (as measured by cord leptin) between Pakistani and White British origin infants and found no evidence that the difference varies by birthweight. Although these findings offer some reassurance against the suggested potential adverse impact of a positive interaction between ethnicity and birthweight,^11^ the fact that there is increasingly robust evidence of greater relative fatness at birth for a given birthweight in South Asian compared with White European populations^2^^,^^3^^,^^7^^,^^19^ still means that if interventions were developed that increased mean birthweight in South Asians, population levels of greater adiposity in South Asians might increase across the life course. Further work is now clearly needed to understand why South Asian children, irrespective of their birthweight, are born with greater fat mass.

Scientists are encouraged and able to use BiB data. Data requests are made to the BiB executive using the form available from study website www.borninbradford.nhs.uk (please click on ‘Science and research’ to access the form). Guidance for researchers and collaborators, the study protocol and the data collection schedule are all available via the website. All requests are carefully considered and accepted where possible.

## Supplementary Material

Supplementary Data
