# Supplementary material for: Do ethnic differences in cord blood leptin levels differ by birthweight category? Findings from the Born in Bradford cohort study
Source: Int J Epidemiol. 2013 Nov 29;43(1):249–54. doi: 10.1093/ije/dyt225 (PMC3937974; doi:10.1093/ije/dyt225)
Supplement: Supplementary Data [file supp_43_1_249__index.html]

Do ethnic differences in cord blood leptin levels differ by birthweight category? Findings from the Born in Bradford cohort study — Supplementary Data 

# Do ethnic differences in cord blood leptin levels differ by birthweight category? Findings from the Born in Bradford cohort study

## Supplementary Data

files

**Files in this Data Supplement:**

- Supplementary Data - doc file
