# Supplementary material for: Do ethnic differences in cord blood leptin levels differ by birthweight category? Findings from the Born in Bradford cohort study
Source: Int J Epidemiol. 2013 Nov 29;43(1):249–54. doi: 10.1093/ije/dyt225 (PMC3937974; doi:10.1093/ije/dyt225)
Supplement: Supplementary Data [file supp_dyt225_ije-2013-04-0378-File003.doc]

**Supplementary Figure 1**

**Correlation between birthweight and leptin by ethnic group** excluding those mothers with a diagnosis of gestational diabetes*

R^2^ White British infants = 0.1933

R^2^ Pakistani infants = 0.1777

*Gestational diabetes was defined according to WHO criteria as either of fasting glucose ≥ 6.1 mmol/L or two hour glucose ≥ 7.8 mmol/L
